# Diffusion imaging in Huntington’s disease: comprehensive review

**DOI:** 10.1136/jnnp-2020-324377

**Published:** 2020-10-08

**Authors:** Carlos Estevez-Fraga, Rachael Scahill, Geraint Rees, Sarah J Tabrizi, Sarah Gregory

**Affiliations:** 1 Huntington's Disease Centre, Department of Neurodegenerative Disease, UCL Queen Square Institute of Neurology, University College London, London, UK; 2 Wellcome Centre for Neuroimaging, University College London, London, UK; 3 Institute of Cognitive Neuroscience, University College London, London, UK

**Keywords:** Huntington's, image analysis, movement disorders, MRI

## Abstract

Huntington’s disease (HD) is a monogenic disorder with 100% penetrance. With the advent of genetic testing in adults, disease-related, structural brain changes can be investigated from the earliest, premorbid stages of HD. While examining macrostructural change characterises global neuronal damage, investigating microstructural alterations provides information regarding brain organisation and its underlying biological properties. Diffusion MRI can be used to track the progression of microstructural anomalies in HD decades prior to clinical disease onset, providing a greater understanding of neurodegeneration. Multiple approaches, including voxelwise, region of interest and tractography, have been used in HD cohorts, showing a centrifugal pattern of white matter (WM) degeneration starting from deep brain areas, which is consistent with neuropathological studies. The corpus callosum, longer WM tracts and areas that are more densely connected, in particular the sensorimotor network, also tend to be affected early during premanifest stages. Recent evidence supports the routine inclusion of diffusion analyses within clinical trials principally as an additional measure to improve understanding of treatment effects, while the advent of novel techniques such as multitissue compartment models and connectomics can help characterise the underpinnings of progressive functional decline in HD.

## Introduction

Huntington’s disease (HD) is a genetic, autosomal dominant disorder caused by a CAG trinucleotide repeat expansion in the Huntingtin gene, encoding for the toxic mutant Huntingtin protein (mHTT). HD is characterised by a triad of neuropsychiatric, cognitive and motor symptoms with onset during early adulthood, progressing to dementia and death within 20 years.^1^

Progressive striatal atrophy, extending to the white matter (WM) and eventually the entire cortex, is the neuropathological hallmark of HD.^2^ In agreement with early histopathology studies, neuroimaging shows that while the striatum is the first brain area to be affected by the HD mutation,^3^ neuronal loss soon spreads to the WM during premanifest stages.

Loss of WM organisation is one of the characteristic features of neurodegenerative disorders.^3^ In HD, there is evidence of early and progressive thinning of myelin sheaths, decreased expression of myelin-related genes and myelin basic protein.^4^ Moreover, oligodendrocytes, the cell subtype that provides myelin to axons in the central nervous system, show increased density in the brains of HD gene carriers before changes in other brain cells are detected, suggesting an early effect of the disease on the myelination process.^5^ Inactivation of mHTT within oligodendrocytes prevents myelin deficiencies and ameliorates behavioural phenotypes in mouse models of HD, possibly due to improved cholesterol metabolism and increased transcription of myelin regulator factor.^6^ However, most experimental evidence about histological abnormalities in HD is based on mouse models.^4^ Therefore, investigating WM microstructure in neurodegeneration in vivo through neuroimaging is critical to the understanding of neurological diseases such as HD.

### Basic concepts in diffusion

Brownian motion, or diffusion, describes the random movement of particles within a fluid. In an isotropic medium, the displacement of molecules tends to follow a Gaussian distribution. However, in the presence of boundaries, such as myelinated fibres, the displacement of molecules tends to occur preferentially in one direction, representing anisotropy.^7^

Diffusion-weighted imaging (DWI) uses specific sequences sensitive to the motion of water molecules.^8^ It is acquired in multiple directions in order to infer direction and coherence of WM tracts using echo-planar imaging, an MRI acquisition technique sensitised to the diffusion of water molecules and which allows imaging of the whole brain in seconds.^8^

Diffusion images reflect the nature of water movement; for example, the darker a voxel (or three-dimensional (3D) pixel) appears within an image, the greater the rate of diffusivity within that voxel. As such, cerebrospinal fluid (CSF) will appear black in diffusion images representing a large net displacement of water molecules causing the MRI signal to decrease rapidly. Conversely, where water movement is restricted, for example, in WM tracts, MRI signal decreases more slowly and voxels appear brighter.

### Models of diffusion

**Figure 1:**
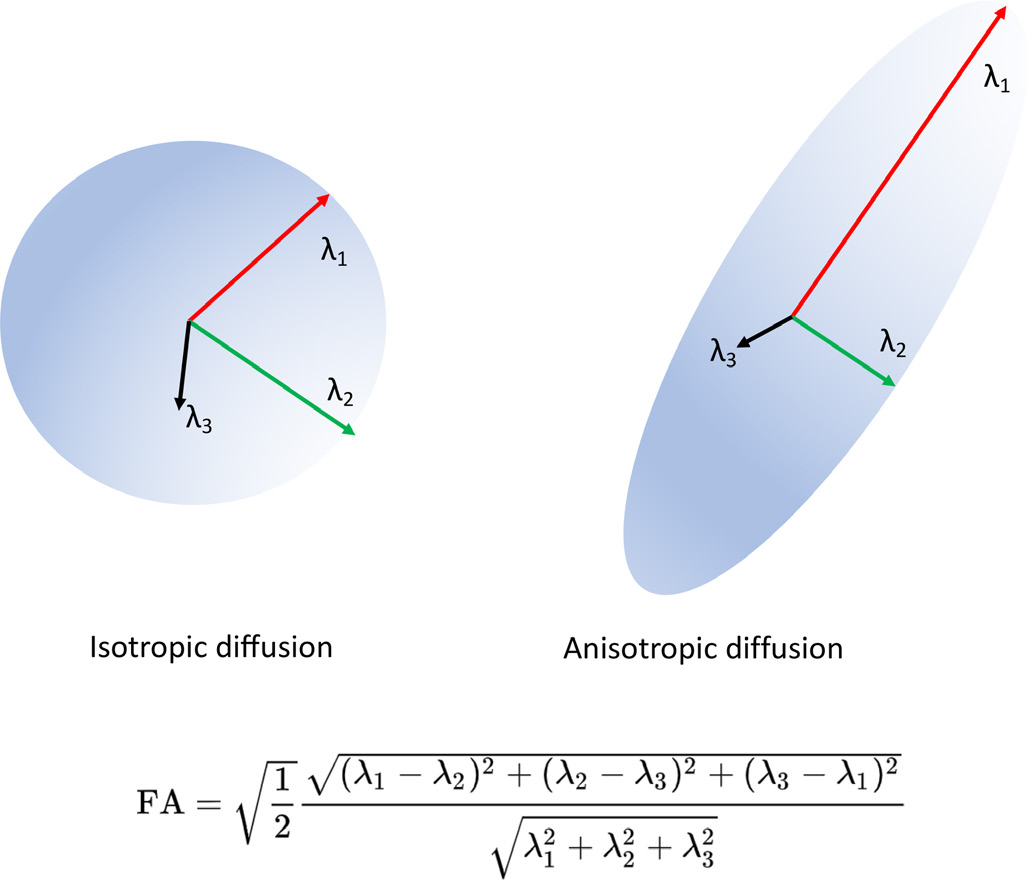
Diagram of diffusion tensor and FA equation. The diffusion tensor is illustrated with its three eigenvectors. An isotropic (FA value close to 0) and an anisotropic (FA value close to 1) diffusion ellipsoids are depicted. The FA equation is also shown. FA, Fractional Anisotropy.

**Figure 2:**
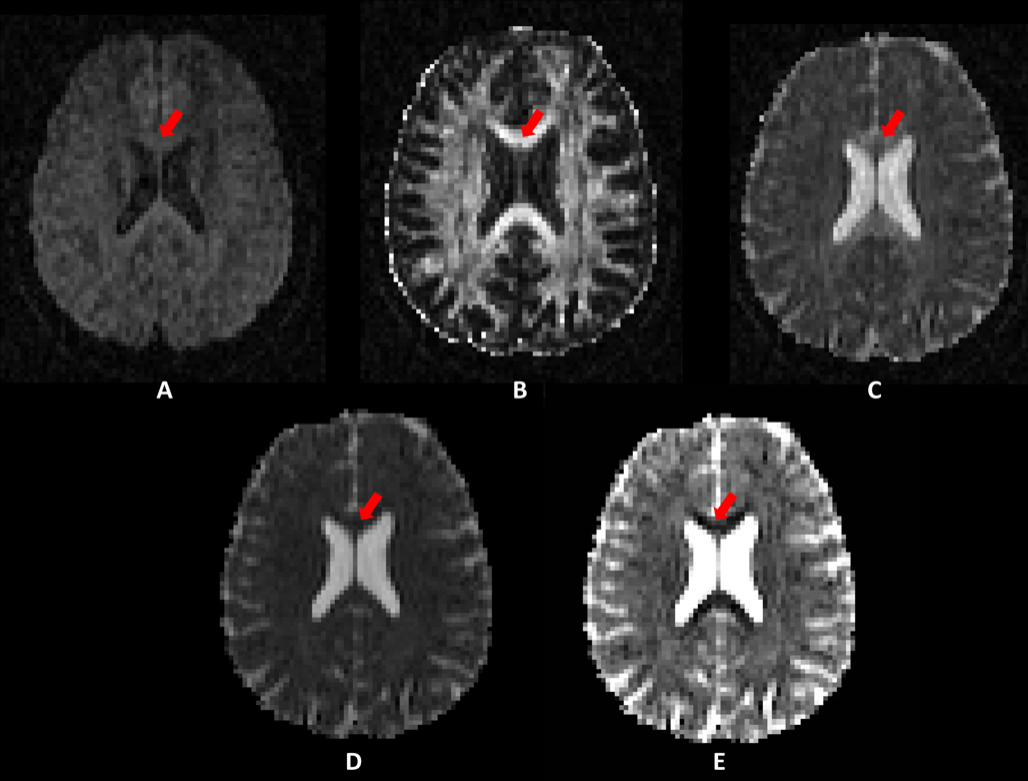
(A)Diffusion weighted imaging raw image; (B) fractional anisotropy map; (C) axial diffusivity map; (D) mean diffusivity map; and (E) radial diffusivity map from one participant. Note the differences in areas with high white matter coherence such as in the corpus callosum (red arrows)DWI, Diffusion-weighted imaging; DTI, Diffusion Tensor Imaging.

Diffusion tensor imaging (DTI) uses a tensor-based model of diffusivity characterised by three vectors (eigenvectors ε1, ε2 and ε3) that represent the direction of diffusion along the three main axes, and three values (the eigenvalues λ_1_, λ_2_ and λ_3_) that represent the magnitude of diffusivity.^7^ This is illustrated in figure 1. The longest eigenvalue (λ_1_) represents axial diffusivity (AD) (figure 2), which is a measure of diffusivity in the direction of the main underlying WM pathway. The two shorter axes (λ_2_ and λ_3_) when averaged constitute radial diffusivity (RD) (figure 2), which represents diffusivity in the pathways perpendicular to the main underlying pathway.^7^ Mean diffusivity (MD) is equal to the average of the three diffusivity vectors (figure 2).^7^ Fractional anisotropy (FA) (figures 1 and 2) is probably the most widely used DTI metric. It is a scalar value that goes from zero when diffusion is completely isotropic, to one when it is anisotropic. Diffusion metrics are used to make statistical inferences regarding underlying WM organisation either between groups or in terms of correlations with a scale to provide, for example, clinical correlates of microstructural disorganisation.^9^

### Data analysis

Once modelled, the resulting diffusion metric maps can be analysed using a series of different techniques according to the clinical question. The most commonly used approaches are whole-brain voxel-wise, region of interest (ROI) and tract-based, which will be discussed further.

#### ROI analysis

**Figure 3:**
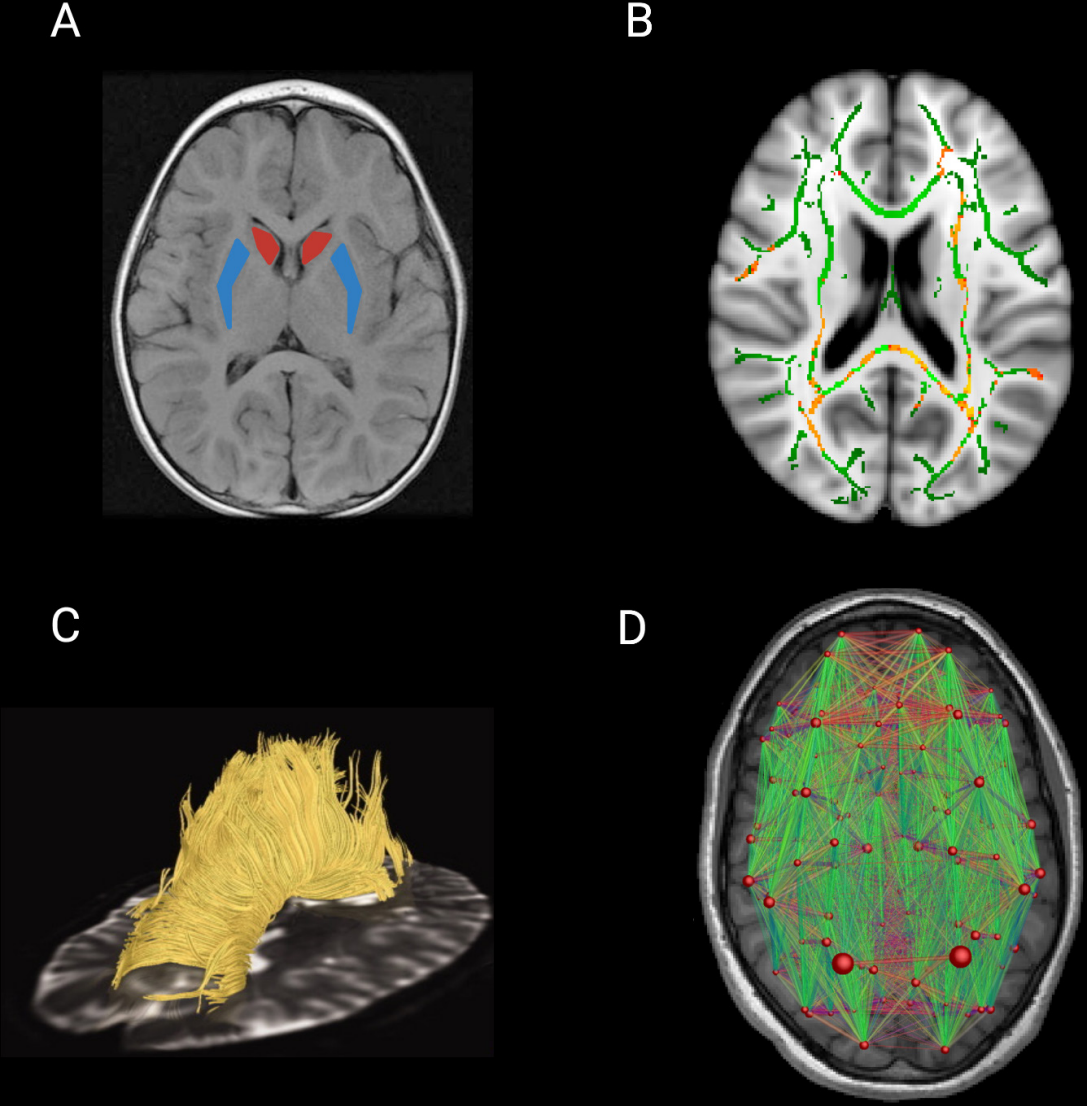
Diffusion imaging analysis techniques. (A) ROI analysis delineating the caudate (red) and the putamen (blue) nuclei. (B) Output of TBSS whole-brain analysis. The FA skeleton is represented in green, overlaid in the Montreal Neurological Institute standard brain template. Significant results are generally represented on a red-yellow scale with brighter colours indicating higher p values. (C) Image representing WM tracts of the CC in a premanifest gene carriers for tract-based analysis (adapted and reproduced with permission from Dumas *et al* 33). (D) Connectivity matrix of the brain with nodes representing brain regions and edges representing WM connections. The size of the nodes represents a given connectivity metric and the size of the edges represents streamline count (adapted and reproduced with permission from Roine *et al* 12). CC, corpus callosum; FA, fractional anisotropy; ROI, region of interest; TBSS, tract-based spatial statistics; WM, white matter.

In ROI analyses (figure 3A), one or several anatomical areas of the brain are selected for investigation based on an a priori hypothesis. Diffusion metrics are extracted from ROIs and then included in statistical analysis. ROIs can either be derived from a structural T1 scan and then moved into the same ‘space’ as the diffusion scan or by applying standard atlases directly onto the diffusion image.^8^

#### Whole-brain analysis

Early whole-brain diffusion studies in HD used a data-driven approach, performing voxel-based analyses of DTI metric maps to examine all brain voxels simultaneously. It is essential to ensure that a specific voxel contains information from the same WM tract for every participant, and voxel-based analyses are prone to misalignment errors. Tract-based spatial statistics (TBSS) (figure 3B) includes a skeletonisation step that enables measurement of WM diffusivity in areas where anisotropy is above a certain level, that is, more coherent. TBSS prevents misalignment avoiding the need for smoothing and has become the standard approach to whole-brain DTI analysis since its initial release. It should also be noted that new models with improved registration are constantly being developed, but as yet are not as widely used as TBSS.^10^

#### Tractography

Tractography is used to measure diffusivity within WM pathways by reconstructing 3D structures of axonal bundles (figure 3C).^11^ It can be applied in cases where there is a clear hypothesis regarding tracts impacted by pathology or in more exploratory, whole-brain, connectome-based analyses. Tractography approaches either assume a single diffusion orientation for WM fibres within a voxel (deterministic tractography) or estimate multiple, possible diffusion orientations within a voxel (probabilistic tractography).^12^ Previous challenges in tract-based studies, such as determining the precise origin and termination of the connections in the cortex, or limited anatomical accuracy, have been overcome in recent years, with huge advances in the field leading to tractography techniques proving to be a powerful tool to study the human brain.^11^

## Diffusion in HD

Here, we present an overview of the literature where DWI has been employed to investigate brain microstructure in HD, providing an insight into HD pathophysiology over the course of the disease.

To look for relevant articles, we performed a comprehensive search for diffusion studies in HD in the PubMed database. We used the following search keywords: Huntington’s disease, Huntington disease, diffusion, diffusion tensor, DTI, white matter microstructure, tract-based spatial statistics and TBSS. The reference lists of the identified articles were also manually reviewed to search for additional papers. The articles were preselected based on their abstracts. The criteria for final selection were studies that provided relevant information about patients with HD premanifest and/or manifest HD in the opinion of the authors. Novel methods and large studies with well-characterised cohorts were prioritised.

## Cross-sectional diffusion studies in HD

### Basal ganglia (BG) and deep WM

**Figure 4:**
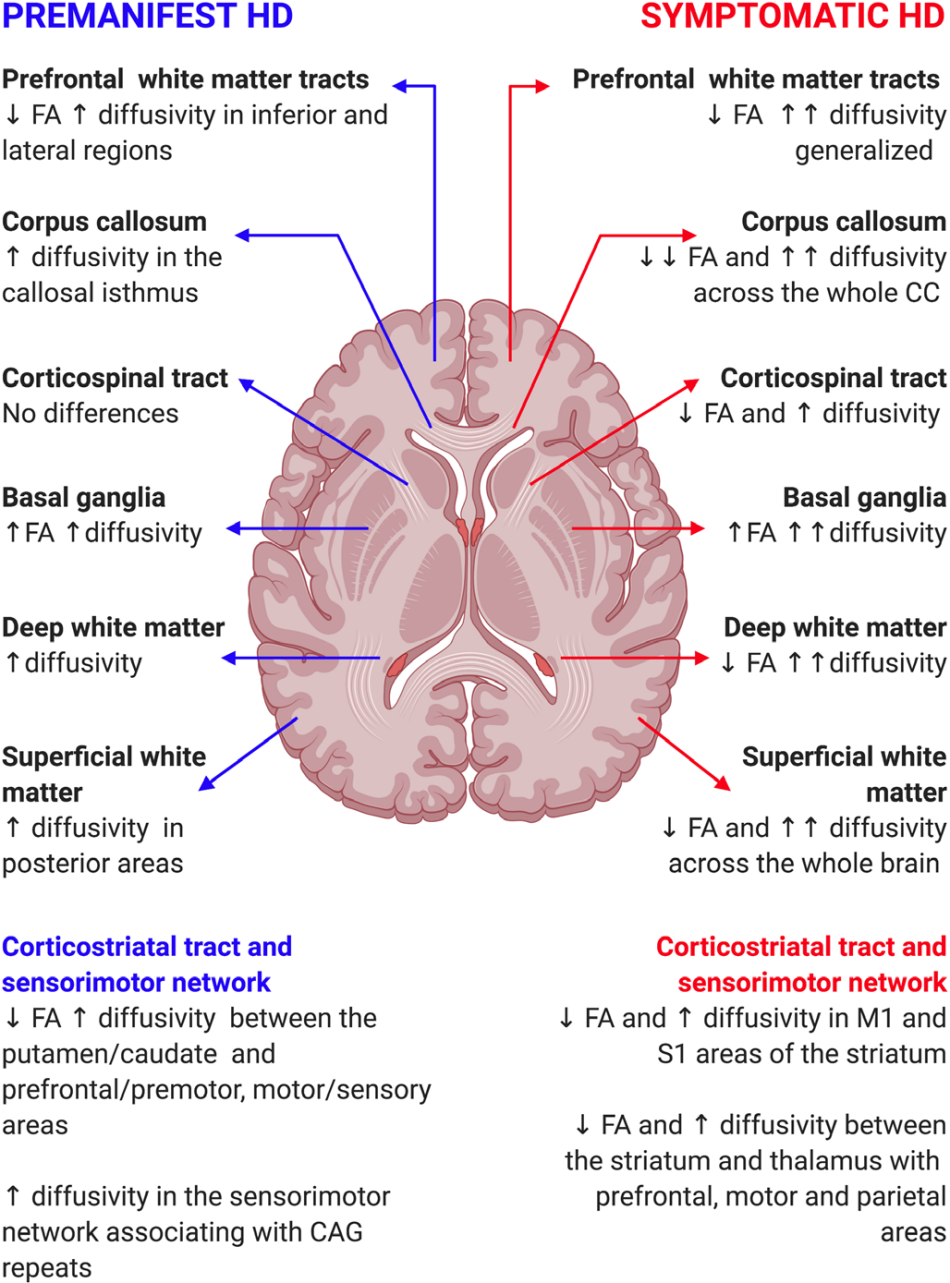
Summary of cross-sectional diffusion studies in HD. ↑, increase; ↓, decrease; ↑↑, marked increase; ↓↓, marked decrease; FA, fractional anisotropy; HD, Huntington’s disease.

While the main focus of diffusion analysis is WM, early whole-brain, voxel-based studies investigated the BG. Diffusion analysis of the BG is difficult to interpret biologically, but such observations are nevertheless of interest, given the prominent involvement of the BG in HD pathology. Evidence of increased MD in the BG in both premanifest (pre-HD) and symptomatic HD is expected due to cell death and blood–brain barrier dysfunction.^13–15^ However, congruent, yet counterintuitive, increases of FA have also been widely reported, suggesting higher levels of organisation despite increased cell loss^13 16–20^ with increased striatal MD and FA in pre-HD, for example, extending to neighbouring areas, such as globus pallidus, accumbens and internal capsules in manifest patients.^13 18^ A study combining whole-brain and BG ROI analyses showed that selective degeneration of specific WM tracts in early HD can lead to decreased dispersion of BG fibres, higher anisotropy values and therefore a paradoxical increase in microstructural organisation despite neural loss.^21^ Similar increases of FA in grey matter (GM) have also been reported in other neurodegenerative diseases, such as multiple sclerosis and Alzheimer’s disease, yet the significance of FA in GM still remains unclear (online supplemental tables S1 and S2 and figure 4).^21^

10.1136/jnnp-2020-324377.supp1 Supplementary data

10.1136/jnnp-2020-324377.supp2 Supplementary data

In addition to subcortical GM, deep WM is also selectively vulnerable to neuronal loss from premanifest stages. For example, increases in diffusivity were present in all studied WM tracts for the baseline visit of the longitudinal PREDICT-HD study, but over time, diffusivity changes became more pronounced in deep subcortical WM areas.^22 23^ Interestingly, early MD increases appear to precede FA reductions, which become apparent as disease progresses.^9 24 25^

### Corticostriatal connections and the sensorimotor network

Degeneration of medium spiny neurons (MSNs) in the striatum is one of the main pathological hallmarks of HD,^26^ with MSNs receiving inputs from cortical areas in a topographically specific manner. Studies show an anterior–posterior organisation of corticostriatal pathway degeneration in manifest HD,^27^ supported by both pathological studies in healthy ageing as well as structural volumetric and histopathological studies in HD.^2 3^ Further evidence of an anterior–posterior gradient has been related to functional connectivity using resting-state functional MRI, which examines the temporal correlation between remote brain areas when the brain is at rest, with decreased structural connectivity in pre-HD relative to controls being associated with an upregulation of functional connectivity in frontal areas and downregulation in occipital regions.^28^

Moreover, as HD progresses, changes appear most notable in sensorimotor and associative corticostriatal pathways^29^ with selectively decreased connectivity between the putamen and prefrontal/motor cortices in pre-HD extending to caudate–parietal cortex connections in manifest HD.^30 31^ Similarly, increased diffusivity in WM tracts connecting the sensorimotor cortex and CC in pre-HD^32^ becomes more evident in manifest HD, extending to thalamic and prefrontal WM pathways.^33^

The sensorimotor network is of particular interest in HD, extensively reported to be altered using clinical, neurophysiological assessments and cortical thinning,^3 34^ in addition to ROI-based and whole-brain DTI analyses.^35–38^ Connectivity between the striatum (putamen and caudate) and motor, somatosensory and premotor areas in the PREDICT-HD study revealed strong associations between disease burden and WM disorganisation. Interestingly, longitudinal studies have shown that striatal volume changes precede changes in DTI measures in the corticostriatal network. However, although reductions in striatal volume are more prominent than DTI changes in participants with pre-HD with lower disease burden, microstructural longitudinal changes are more marked in patients with pre-HD closer to disease onset, suggesting that microstructural changes occur more rapidly and begin later than striatal volume loss.^39^

Similarly, in the TrackOn-HD cohort, a pattern of volume loss, altered cortical thickness and disturbed diffusivity in the sensorimotor network was only present in HD gene carriers compared with controls and was associated with CAG repeat length.^40^ In the same cohort, a dimension–reduction approach revealed three independent patterns of diffusivity across 32 pathways. The first showed widespread increased RD and AD with decreased FA, while the second one showed increased FA and AD in the sensorimotor network only; the third revealed reduced RD and increased FA in connections between prefrontal cortex, thalamus and caudate. All three patterns were more pronounced in gene carriers, suggesting that HD does not necessarily damage connectivity networks but, in fact, accentuates existing ones.^41^

Finally, as an essential component of the sensorimotor network, carrying motor impulses from the cortex to the spinal cord, the corticospinal tract (CST) has also been examined using diffusion imaging. CST conduction essentially remains normal in HD and, as such, there is little evidence of DWI changes in pre-HD.^42 43^ However, selective degeneration has been shown in manifest patients, potentially related to clinical diagnosis, which centres exclusively on motor symptoms.^42^

### Corpus callosum (CC)

The CC is the primary commissural region of the brain integrating cognitive function between the two hemispheres.^44^ It is a highly myelinated structure with marked coherence and is an ideal target for investigation using DTI. In pre-HD, the CC is associated with cognitive and motor deficits^27 45^ with decreased thickness and increased diffusivity limited to the callosal isthmus, and which becomes more pronounced in those closer to disease onset.^46^ In contrast, manifest HD gene carriers display decreased width across the whole structure of the CC alongside widespread alterations in FA, RD and AD, indicative of a breakdown of callosal fibres.^44 46–48^ The progression of both structural volumetric and DTI abnormalities suggests a temporal evolution of changes, with FA being affected first followed by RD and finally AD.

### Prefrontal lobe

The prefrontal cortex is highly connected to the BG influencing cognitive processes and in turn, the cognitive symptoms evident in HD.^49^ Prefrontal WM tracts in pre-HD show increased FA and RD in inferior and lateral regions.^37^ These changes correlate with executive function performance in those closer to disease onset and increased in gene carriers with higher disease load.^36^ Although not specifically assessed in manifest gene carriers, whole-brain studies show marked increases in MD and less pronounced changes in FA in WM tracts connecting with the prefrontal cortex.^9^

### Cerebellum

Cerebellar atrophy is often reported in patients with juvenile-onset HD, and neuropathological studies show neuronal loss and decreased Purkinje cell density in patients with adult-onset manifest HD.^2^ Although whole-brain approaches show only minor decreases in cerebellar diffusivity,^50^ a ROI study evidenced widespread diffusion increases in AD, MD and RD both in GM and WM in manifest HD that correlate with motor and psychiatric symptoms.^51^

In summary, evidence from diffusion studies shows a robust pattern of structural connectivity change in HD. Microstructural alterations begin during premanifest stages in BG^13 18 52^ and deep WM.^53^ As disease progresses, changes in deep brain regions increase in magnitude,^44 53^ while more superficial areas and connections become affected.^30 54^ Patients with manifest HD show more extensive WM microstructural alterations, with changes extending from deep brain regions towards the cortex, indicating a progressive, topographically specific WM degeneration in HD.

## Longitudinal diffusion studies in HD

Few studies have assessed longitudinal diffusivity changes in HD (online supplemental table S3). Harrington and colleagues showed a significant MD increase in the superior fronto-occipital fasciculus (one tract out of a possible 16), in pre-HD over a period of 24 months.^55^ However, another study that selectively evaluated corticostriatal connectivity in a larger subsample of the same cohort found significant changes in at least one DTI metric for all tracts examined.^39^ In both studies, these changes were especially pronounced in participants closer to disease onset.^39 55^ Moreover, pre-HD gene carriers showed increased MD in the BG and CC after one year when using a hypothesis-driven ROI-based approach; these findings were not confirmed, however, when using an unbiased whole-brain analysis.^56^

10.1136/jnnp-2020-324377.supp3 Supplementary data

Although most studies have not reported evidence of longitudinal change prior to motor onset,^57 58^ manifest gene carriers display consistent decreases in FA in areas with high WM coherence such as the CC and corona radiata.^59^ ^35 60^ Furthermore, the IMAGE HD cohort revealed longitudinal increases in FA in the BG of symptomatic patients with HD after 30 months,^57^ while the PADDINGTON cohort showed increases in diffusivity metrics exclusively in the BG in a similar population.^61^

In conclusion, detectable longitudinal changes in manifest HD affect similar areas to those seen in cross-sectional studies; these are only subtle in pre-HD.^39 55^ Studies with larger cohorts and longer follow-up periods are needed to increase sensitivity for the detection and characterisation of the pathophysiological consequences of changes in WM organisation.

## Clinical implications of diffusion studies

Many studies examine changes in WM organisation in relation to clinical and neuropsychological functions. These are generally presented as correlations between diffusion metrics and, for example, disease burden or Unified Huntington's Disease Rating Scale (UHDRS) Total Motor Score. While not causal in terms of the relationships they measure, these analyses indicate how different domains of the HD phenotype are related to changes in particular WM pathways.

### Correlates with motor deficits

In HD, clinical diagnosis is based solely on motor symptoms, which have a characteristic evolution of hyperkinetic movements followed by parkinsonism. Correlation studies have shown that diffusivity in corticostriatal connections between the putamen and prefrontal as well as primary motor cortex are associated with scores in motor scales in HD gene carriers.^30^ In addition, there are robust associations between diffusion in the CC and movement dysfunction, with significant correlations between MD in the CC and tapping variability, sustained tongue force^33^ and UHDRS Total Motor Score,^27^ suggesting a contribution of this structure to the motor deficits in HD. Finally, diffusivity in the cerebellum is also significantly correlated with motor dysfunction in HD^51^ possibly through the connectivity between this structure and the BG.^62^

### Correlates with cognitive scales

Although cognitive dysfunction in HD involves brain regions across various cognitive domains, cognitive measures such as the Symbol Digit Modalities Test and Stroop Word Reading are sensitive to disease progression in HD.^3^ These scales show associations in pre-HD and manifest HD with diffusivity in the putamen–frontal tract,^30^ the CC and with FA in total WM.^9 33 44^ In addition, a global composite score encompassing nine cognitive domains is significantly correlated with the strength of connections between left and right motor–occipital–parietal modules, as well as with intrahemispheric corticostriatal and intramodular left frontocingulate connections in pre-HD.^63^

### Correlates with neuropsychiatric scales

Despite neuropsychiatric symptoms being one of the core features of HD, few studies have specifically investigated associations between psychiatric scales and microstructural changes.^64 65^ Interestingly, scores in depression scales are associated with decreased FA in the cerebellum, frontal and cingulate cortex, and insula before any volume loss can be identified, maybe indicating an earlier detectable neuronal damage in WM microstructure than in volumetric MRI in symptomatic patients.^64^ However, while apathy scores correlate with corticostriatal connectivity,^66^ depressive symptoms are associated with the connections between cingulate, orbitofrontal, precuneus, caudate and thalamus, but not the corticostriatal circuit, suggesting specific neuroanatomical substrates for the different neuropsychiatric symptoms present in HD.^65^

## Advanced techniques in diffusion imaging

### Diffusion imaging in GM

Although DWI is generally used in WM to infer the strength and coherence of WM tracts, alterations in diffusivity in GM have been shown since early voxel-based studies with increased MD and FA in the BG of HD gene carriers.^13 21^

In addition, recent evidence has evaluated MD in the cortex of patients with different conditions through surface-based intracortical MD. This method determines the MD values halfway between pial and WM surface through coregistration between MD maps and T1 scans, followed by partial volume correction to avoid the inclusion of voxels containing CSF.^67^

This technique has shown extensive cross-sectional and longitudinal increases in cortical MD in a range of neurological disorders such as Alzheimer’s disease,^68 69^ Parkinson’s disease^67^ and frontotemporal dementia.^70^ In HD, a recent study using the same methodology demonstrated that cortical MD is increased even in the absence of cortical atrophy since premanifest stages of the disease. Moreover, there was a marked increase with disease progression that correlated with disease burden and different clinical scales.^71^ Although methodological considerations may limit the interpretation of these results, these findings suggest the presence cortical microstructural degeneration in HD possibly related to cell membrane breakdown since early stages of the disease.^68^

### Newer modelling methods, beyond the tensor

Despite the presence of a number of tissue types within each voxel, DTI models diffusivity uniformly and therefore cannot differentiate the diffusivity characteristics associated with each microstructural tissue component.^7^ Advanced techniques have been developed, which are able to estimate diffusivity associated with different tissue types, in turn providing more information regarding the underlying biological properties associated with microstructural change.^72^

Neurite orientation dispersion and density imaging (NODDI) has recently been used in HD cohorts to characterise microstructures more fully.^73–75^ NODDI uses a three-tissue compartment model whereby each tissue element is independently estimated according to its proposed diffusion properties. The three components are intraneurite, extraneurite and a CSF component. In the case of WM, neurite measures represent underlying axons (as opposed to dendrites in GM). Output metrics for NODDI include Orientation Dispersion Index (ODI), which refers to the organisation of neurites; Neurite Density Index (NDI) related to the density of axons, and the free water fraction.^72^ These metrics can be used alongside DTI measures to gain a greater understanding of the underlying biological properties of WM microstructural changes.^72^ In a cohort of HD gene carriers, on average 23.6 years before clinical diagnosis, there were no differences in any DTI or NODDI metric when compared with healthy controls,^73^ but in those closer to motor onset from the TrackOn-HD cohort, extensive reductions in NDI correlated with disease burden and motor dysfunction, while reduced ODI in the internal and external capsules was suggestive of some degree of selective pruning of WM fibres early in the disease.^74^ These widespread decreases in NDI, combined with increased diffusivity, persist in manifest HD gene carriers.^75^

Other advanced techniques include quantitative magnetisation transfer, which nominally tracks demyelination, by characterising the macromolecular proton fraction^76^ and correlates with increases in diffusivity in HD.^77^ Alternatively, the isotropic volume fraction measures extracellular fast-moving water, a potential proxy measure of WM atrophy, which has been shown to be sensitive to disease-related WM change in corticostriatal pathways.^78^

While newer diffusion modelling techniques suggest greater sensitivity to HD pathology-related change, they require both further in vivo investigation in large, longitudinal studies and validation with postmortem pathological findings.

### Connectomics, a different approach to diffusion analysis

In addition to newer modelling techniques, there have also been significant advances in the methods used to analyse diffusion metrics. ROI and whole-brain voxel-based analyses are useful in terms of identifying where microstructural changes occur and continue to be implemented routinely. However, approaches such as network-based analyses of brain connections, or connectomics (figure 3D), can provide additional information regarding the interaction of neural connections across the brain.^79^

Pre-HD gene carriers display selective degeneration of subcortical hub brain areas, that is, those that are most highly connected, extending to those in the cortex as the disease progresses.^79^ There is also an apparent hierarchical pattern of vulnerability associated with WM pathway length, such that longer connections are affected earlier.^63^ In line with previous findings,^30^ corticostriatal connections first show decreased connectivity followed by interhemispheric and then intrahemispheric connections.^63^ Interestingly, corticostriatal and interhemispheric connections are associated with a synaptic gene profile, while intrahemispheric tracts are associated with metabolic genes.^80^ The breakdown of long-range connectivity versus maintenance of localised connectivity increases was evident during both pre-HD and early manifest HD stages, correlating with clinical scales. Importantly, as indicated previously, hub functionality proceeded to diminish over time.^81^

These studies are consistent with pathological reports supporting the transneuronal diffusion of the mHTT protein, which may underscore early WM degeneration in densely connected networks.^1^ Further supporting this hypothesis, in a sample of manifest HD and healthy controls, using a network diffusion model predicted neuron-to-neuron distribution of pathology, suggesting that the healthy connectome explained the pattern of atrophy in HD, especially in areas that were affected earliest.^82^ This is in agreement with tractography studies where patterns of connectivity in controls determine decay in WM coherence in HD.^41^

## Diffusion metrics as biomarkers for clinical trials

Several studies have investigated the potential utility of diffusion measures as markers of drug efficacy in clinical trials. The Track-HD cohort comprised pre-HD, symptomatic HD and controls followed up annually over three years, investigating quantifiable endpoints to inform future disease-modifying clinical trials with a particular focus on imaging metrics.^3^ DWI was performed at three of the four participating sites and showed both widespread decreases in FA and widespread increases in MD in patients with manifest HD; increased MD was localised to the CC, external capsule and inferior longitudinal fasciculi in pre-HD.^83^

Similarly, the PADDINGTON study investigated changes in brain structure in early HD across a series of short intervals.^61^ This study was primarily purposed to assist the design of trials using multiple study sites with heterogeneous acquisition parameters. The authors also compared effect sizes of volumetric measures with those from diffusion MRI to determine which modalities performed better in terms of classifying participants as HD or controls. Here, there were significant differences in all DTI metrics in the putamen, caudate, total WM and CC when compared with controls. Moreover, although the largest effect sizes were for macrostructural metrics, diffusion in the putamen and caudate nuclei showed excellent sensitivity with increased diffusivity and increased FA.^24^ Further results from PADDINGTON have shown that, assuming a disease-modifying treatment effect of 50% over a 15-month period, a sample size of less than 70 patients per treatment arm would be required for DTI metrics to detect efficacy, while the most sensitive clinical scale for HD would require twice as many patients.^61^

In addition, surface-based cortical MD has shown excellent sensitivity for the detection of cortical changes in other neurodegenerative disorders, with moderate-to-high effect sizes^70^ supporting its inclusion in clinical trials if further HD studies confirm these findings.

Although most studies include both clinical and diffusivity metrics, a statistical comparison of effect sizes has not been routinely performed. However, DTI metrics consistently outperform clinical measures both in isolation (eg, cognitive) or when combined in composite scores from premanifest stages.^13 17 84^

Although DWI is still some way from being integrated as a diagnostic tool for neurodegenerative processes, diffusivity can be used as a secondary outcome in clinical trials to aid the evaluation of drug efficacy and safety. For example, laquinimod is an immunomodulatory drug that targets the central nervous system and has been evaluated in patients with HD. Interestingly, although the primary outcome of the human trial in HD with laquinimod did not show disease-modifying effects, the drug had a significant positive impact on imaging biomarkers.^85^ These findings were also in agreement with results obtained in HD mouse models showing improvements in WM microstructure as well as rescuing atrophy in different brain regions, including the striatum.^85 86^

## Limitations and applicability of diffusion imaging in HD

In this review, we have focused on the most recent DTI studies in HD. It should be noted, however, that there are meaningful differences in terms of study design, data acquisition and analysis technique, which limit comparisons between studies.

In addition to the limitations that are inherent to the technique, such as the presence of crossing fibres within the same voxel or the inability to detect directionality of the axons,^8^ HD has some characteristics that may affect the reliability of DWI studies. The presence of chorea may cause movement artefacts in images, but this is generally only a limitation in a minority of patients with uncontrollable movements of large amplitude and can be improved with motion-correction software after image acquisition.^87^ Multicentric imaging studies are an efficient way to group patients with rare diseases such as HD; however, differences in MRI scanner or software potentially limit the sensitivity of analysis to detect changes,^8^ although these differences can be accounted for using multilevel modelling procedures or constraining data from various sources.^88^ In addition, diffusivity measures such as FA, MD, AD and RD have longitudinal intraclass correlation coefficients above 0.80, indicating good reliability over time.^88^ Thus, despite methodological concerns, there is robust evidence in HD to suggest that multicentric imaging studies are feasible and reproducible with good test–retest reliability.^3 87^

## Conclusions

In summary, degeneration of WM microstructure in HD occurs at a relatively early point during the disease course with a temporarily specific pattern that is also associated with meaningful clinical outcomes. At a cross-sectional level, there is a centrifugal pattern of degeneration with deeper brain areas being affected prior to superficial ones^9 24 53^; this is in agreement with histological studies.^2^ Sensorimotor regions, areas more densely connected, and longer tracts tend to be affected first, resulting in a loss of integration of neural networks in HD.^28 63 79^

Over the last 15 years there have been huge advances in the field of structural connectivity and the investigation of WM organisation. From whole-brain voxel-based studies analysing standard DTI metrics^15^ to TBSS,^46 75^ which improves the alignment of the different WM tracts.^10^ Furthermore, hypothesis-driven ROI studies restrict the analyses to relevant areas, whereas tractography has allowed us to visualise in vivo the anatomical WM tracts with increasing precision.^8^ Finally, different tissue compartments within a voxel can be modelled using novel techniques such as NODDI,^72^ while the field of connectomics has provided a radically different approach to the analysis of the brain as a network using graph theory.^79^

In the 2009 review entitled ‘MRI of Huntington’s disease: preparing for clinical trials’, Klöppel *et al* mentioned that ‘It is still too early to decide on the usefulness of DWI to either understand the pathophysiology of HD or in the preparation of treatment trials’.^89^ Ten years later, we have a much better understanding of the biological and clinical correlates of diffusion MRI and DWI is a routine exploratory sequence in trials with neurodegenerative populations.
