# Supplementary material for: Diffusion imaging in Huntington’s disease: comprehensive review
Source: J Neurol Neurosurg Psychiatry. 2020 Oct 8;92(1):62–9. doi: 10.1136/jnnp-2020-324377 (PMC7803908; doi:10.1136/jnnp-2020-324377)
Supplement: Supplementary data [file jnnp-2020-324377supp001.pdf]

Table S1 – ROI and whole-brain cross-sectional studies*

		Participants			ROI analysis				Whole-brain analysis				
**Author**	**Year**	**Controls**	**PreHD**	**HD**	**FA**	**MD**	**AD**	**RD**	**FA**	**MD**	**AD**	**RD**	**Threshold**
**Mascalchi**	2004	21	2	19	N/A	HDGC:↑ in BG, deep WM and whole WM	N/A	N/A	N/A	N/A	N/A	N/A	p<0.05
**Reading**	2005	7	7	Not included	N/A	N/A	N/A	N/A	PreHD: ↓ in the superior frontal, post-central and pre-central WM	N/A	N/A	N/A	p<0.005 uncorrected, k>50 voxels
**Rosas**	2006	29	15	17	PreHD and HD: ↓ in CC and ↑ IC and BG	N/A	N/A	N/A	PreHD and HD: ↑ in BG ↓ in CC, EC, cerebral peduncles, brainstem, thalamus, subcortical WM	PreHD and HD: ↑ in BG	N/A	N/A	p<0.05
**Kloppel**	2008	20	25	Not included	N/A	PreHD: ↓ in BG	N/A	N/A	N/A	N/A	N/A	N/A	p<0.001
**Douaud**	2009	10	Not included	14	HD: ↑ in BG	HD: ↑ in BG	N/A	N/A	HD: ↑ in BG	HD: ↑ in BG, CR, subcortical WM	N/A	N/A	<u>WHOLE-BRAIN</u> p<0.01 FDR corrected for the MD maps p<0.05 FDR corrected for FA maps <u>ROI</u> p<0.05
**Mandelli**	2009	25	15	9	N/A	HD: ↑ in BG PreHD: ↓ in BG	N/A	N/A	N/A	N/A	N/A	N/A	p<0.05

**Della Nave**	2010	15	1	14	N/A	N/A	N/A	N/A	HDGC: ↓ in CC, fornix, EC, IFOF, ILF and ↑ in supralenticular frontal WM	HDGC: ↑ areas with ↓ FA plus AF and CP	HDGC: ↑ in areas with ↓ FA	HDGC: Similar to MD	p<0.05 TFCE corrected
**Sritharan**	2010	17	Not included	18	N/A	HD: ↑ in BG, thalamus and CC	N/A	N/A	N/A	N/A	N/A	N/A	p<0.005 Bonferroni corrected
**Stoffers**	2010	25	39	Not included	N/A	N/A	N/A	N/A	PreHD: ↓ widespread	PreHD: ↑ widespread	PreHD: ↑ thalamic radiations, IC, ED	PreHD: ↑ widespread	p<0.05
**Rosas**	2010	40	19	21	PreHD and HD: ↓ in CC	N/A	PreHD and HD: ↑ in CC	PreHD and HD: ↑ in CC	N/A	N/A	N/A	N/A	p<0.05 Bonferroni-corrected
**Bohanna**	2011	17	Not included	10	HD: ↓ CC, fornix, EC, CR and SS	HD: ↑ same as ↓ FA plus IC and cerebral peduncles	HD: ↑ CR, IC, EC, fornix	HD: ↑ CC, fornix, EC, IC, CR, SS, cingulum, SLF	N/A	N/A	N/A	N/A	p<0.05 corrected for multiple comparisons cluster forming threshold of t>3.
**Di Paola**	2012	34	17	17	N/A	N/A	N/A	N/A	PreHD: ↓ in CC<br>HD: ↓ in CC	N/A	PreHD: No differences<br>HD: ↑ in CC	PreHD: ↑ in CC<br>HD: ↑ in CC	p<0.05 TFCE corrected
**Hobbs**	2013	40	Not included	61	HD: No differences	HD: ↑ in BG	HD: ↑ in BG	HD: ↑ in BG	HD: ↑ in BG in HD ↓ in WM	HD: ↑ widespread	HD: ↑ widespread	HD: ↑ widespread	<u>WHOLE-BRAIN</u> p<0.001<br><u>ROI</u> p<0.05
**Delmaire**	2013	24	Not included	27	N/A	N/A	N/A	N/A	HD: ↑ in BG and ↓ in frontal lobe, CR	HD: ↑ widespread	N/A	N/A	p<0.05 FWE corrected

**Georgiou-Karistianis**	2013	36	35	36	PreHD and HD: ↑in BG	PreHD and HD: ↑in BG	N/A	N/A	N/A	N/A	N/A	N/A	p<0.05
**Sanchez-Castaneda**	2013	30	17	12	PreHD: ↑ in BG<br>HD: ↑ in BG and ↓ in thalamus and hippocampus	PreHD and HD: ↑ MD in BG, hippocampus	N/A	N/A	N/A	N/A	N/A	N/A	p<0.05 Bonferroni corrected
**Matsui**	2014	34	53	Not included	PreHD: No differences	PreHD: No differences	PreHD: No differences	PreHD: ↑ orbitofrontal and a inferior frontal lobe region	N/A	N/A	N/A	N/A	p<0.05 FDR corrected
**Novak**	2014	21	17	19	N/A	N/A	N/A	N/A	HDGC: ↓ CC, SLF, ILF, IC, EC, cingulum	HDGC: ↑ CC, ILF, SLF, IC, EC, cingulum, AF and CP	N/A	N/A	p<0.05 FWE corrected
**Phillips**	2014	50	25	25	PreHD: No differences<br>HD: ↓ in deep WM	N/A	PreHD: ↑ in deep WM<br>HD: ↑↑ in deep WM	PreHD: ↑ in deep WM<br>HD: ↑↑ in deep WM	N/A	N/A	N/A	N/A	p<0.05 FDR corrected
**Odish**	2014	24	22	10	N/A	N/A	N/A	N/A	PreHD: No differences total WM<br>HD: No differences in total WM	PreHD: No differences in total GM or total WM<br>HD: ↑ in total GM and total WM	PreHD: ↑ in total WM, no differences in total GM<br>HD: ↑ in total GM and total WM	PreHD: No differences in total GM or total WM<br>HD: ↑ in total GM and total WM	p≤0.001 Bonferroni corrected
**Rees**	2014	12	Not included	22	HD: ↓ in cerebellar GM	HD: ↑ in cerebellar GM and WM	HD: ↑ in cerebellar GM and WM	HD: ↑ in cerebellar GM and WM	N/A	N/A	N/A	N/A	p<0.05

**Gregory**	2015	36	Not included	48	HD: ↓ in CC and brainstem	N/A	HD: ↑ in CC, CR, EC, IC, PTR, SLF, SS	HD: ↑ in CC, CR, EC, IC, SLF, brainstem	N/A	N/A	N/A	N/A	p<0.05 FDR corrected
**Syka**	2015	14	Not included	14	HD: No differences	HD: No differences	N/A	N/A	N/A	N/A	N/A	N/A	p<0.05 FDR corrected
**Faria**	2016	79	194	Not included	PreHD: No differences	PreHD: ↑ in deep WM, corticostriat al tract	PreHD: ↑ in deep WM	PreHD: ↑ in deep WM and corticostriatal trac	PreHD: No differences	PreHD: ↑ in PTR, CC and occipital WM	Same as MD	Same as MD	p<0.05 FWE corrected
**Harrington**	2016	67	37	Not included	PreHD: ↓ in all tracts	PreHD: ↑ in all tracts	PreHD: ↑ in all tracts	PreHD: ↑ in all tracts	N/A	N/A	N/A	N/A	p<0.05 FDR corrected
**Muller**	2016	32	Not included	34	N/A	N/A	N/A	N/A	HD: ↑ in BG and ↓ in CC, EC, IC and thalamus	N/A	HD: ↑ in BG and dorsal WM	HD: ↑ in BG and dorsal WM	p<0.05 FDR corrected
**Gorges**	2017	13	12	Not included	N/A	N/A	N/A	N/A	PreHD: No differences	N/A	PreHD: No differenc es	PreHD: No difference s	p<0.05 FDR corrected
**Saba**	2017	11	12	11	N/A	N/A	N/A	N/A	PreHD: No differences HD: ↓ in CC, EC, IC, ATR, PTR, SLF, ILF, IFOF	N/A	N/A	N/A	p<0.05 FWE corrected
**Gregory**	2020	20	20	40	N/A	N/A	N/A	N/A	HDGC: ↓ widespread	HDGC: ↑ in CC	HDGC: ↑ in subcortic al WM	HDGC: ↑ in CC	p<0.05 TFCE corrected
**Sweidan**	2020	11	Not included	13	N/A	N/A	N/A	N/A	HD: ↓ widespread	HD: ↓ widespread	N/A	N/A	p<0.05 TFCE corrected

*Only bilateral differences noted. HDGC used when manifest HD and preHD were grouped together in a single group

AD: Axial diffusivity, AF: Arciform Fibers, ATR: Anterior Thalamic Radiations, CC: Corpus Callosum, CP: Cerebral Peduncles, CR: Corona Radiata, CST: Corticospinal Tract, DTI: Diffusion Tensor Imaging, FA: Fractional Anisotropy, IC: Internal Capsule, EC: External Capsule, HD: Huntington's Disease, HDGC: Huntington's disease gene-carriers, IFOF: Inferior Fronto Occipital Fasciculus, ILF: Inferior Longitudinal Fasciculus, MD: Mean Diffusivity, preHD: Premanifest Huntington's Disease, PTR: Posterior Thalamic Radiations, RD: Radial Diffusivity, SS: Sagittal Strattum, SLF: Superior Longitudinal Fasciculus, SFOF: Superior Fronto Occipital Fasciculus, UNC: uncinate fasciculi.
