# Supplementary material for: Diffusion imaging in Huntington’s disease: comprehensive review
Source: J Neurol Neurosurg Psychiatry. 2020 Oct 8;92(1):62–9. doi: 10.1136/jnnp-2020-324377 (PMC7803908; doi:10.1136/jnnp-2020-324377)
Supplement: Supplementary data [file jnnp-2020-324377supp002.pdf]

Table S2 – Cross-sectional tractography studies

Author	Year	Controls	PreHD	HD	Conclusions
Bohanna	2011	14	Not included	12	HD: Corticostriatal connections show an anterior-posterior topographical organization.
Dumas	2012	28	27	16	PreHD: ↑ MD in WM pathways of the sensory motor cortex and CC<br>HD: Extensive ↑ MD in HD
Marrakchi-Kacem	2013	15	Not included	15	HD: ↓ connectivity from caudate to parietal and frontal areas and from putamen to temporal, parietal and frontal regions.
Philips	2014	50	25	25	PreHD: ↓ FA and ↑ AD and ↑ RD in most deep WM tracts preHD<br>HD: Same findings as in preHD plus additional deep WM tracts for each DTI metric
Poudel	2014	35	36	35	PreHD: Decreased connectivity between putamen and prefrontal/motor cortex<br>HD: Decreased connectivity between prefrontal/motor/parietal cortices with caudate/putamen.
Matsui	2015	65	146	Not included	PreHD: Altered diffusivity in all four studied tracts in the prefrontal lobe in the high CAP group, only two tracts in the medium CAP group and none in the lower CAP group
Novak	2015	18	17	14	PreHD: No changes<br>HD: Altered connectivity between basal ganglia and cortex
Phillips	2015	50	25	25	PreHD: No changes in the CST<br>HD: ↓ FA and ↑ AD and ↑ RD in the CST
Muller	2016	32	Not included	34	HD: Diffusivity not associated with functional connectivity in the motor or thalamo-cortical networks
Orth	2016	112	96	35	HDGC: Sensorimotor performance explained by a pattern of GM volume and diffusivity in sensorimotor WM tracts linked to CAG repeat length.<br>Two further components are common between controls and HD, indicating natural variation of patterns already present in healthy subjects.

Gorges	2017	13	12	Not included	PreHD: No differences in diffusivity before disease onset in CST or thalamo-cortical tracts
McColgan	2017	81	70	Not included	PreHD: Negative correlations between depression scores and connectivity between cingulate, orbitofrontal, precuneus, caudate and thalamus. Positive correlations with functional connectivity in cingulate, orbitofrontal, precuneus and parahippocampal regions.
McColgan	2017	66	64	Not included	PreHD: The properties of WM in controls are associated with increases in functional connectivity in HD. Strong connections in anterior regions are increased while strong connections in posterior regions are decreased in preHD.
Shaffer	2017	70	191	Not included	PreHD: Cross sectional and longitudinal differences in all DTI metris in the connections between PMC with putamen and caudate. Differences present only in MD and AD in the connections between M1 and S1 with putamen.
Gregory	2018	79	61	13	HDGC: Three patterns of diffusivity common to HDGC and controls explain the majority of the variability in diffusion and are accentuated in HD.
Rosas	2018	37	31	38	PreHD: ↓ FA and ↑ RD in selected tracts only in the group closer to disease onset. Changes in RD correlated with cognitive performance<br>HD: Widespread ↓ FA, ↑ AD and ↑ RD. Increases in AD associated with cortical thinning
De Paepe	2019	35	22	24	HDGC: ↓ FA and ↑ MD in most corticostriatal tracts

AD: Axial diffusivity, CAP: CAG age product, CST: Corticospinal tract, FA: Fractional Anisotropy, GM: Grey Matter, HD: Huntington's disease, M1: Primary Motor Cortex, MD: Mean diffusivity, PMA: Premotor area, RD: Radial diffusivity, S1: Primary Sensory Cortex, WM: White matter
