# Supplementary material for: Diffusion imaging in Huntington’s disease: comprehensive review
Source: J Neurol Neurosurg Psychiatry. 2020 Oct 8;92(1):62–9. doi: 10.1136/jnnp-2020-324377 (PMC7803908; doi:10.1136/jnnp-2020-324377)
Supplement: Supplementary data [file jnnp-2020-324377supp003.pdf]

Table S3 – Longitudinal DTI studies

			Participants			ROI analysis				Whole-brain analysis				
**Author**	**Year**	**Follow-up (months)**	**Con trols**	**PreHD**	**HD**	**FA**	**MD**	**AD**	**RD**	**FA**	**MD**	**AD**	**RD**	**Threshold**
Weaver	2009	12	7	4	3	HDGC: ↓ in CC	N/A	N/A	N/A	HDGC: ↓ IC, CC, CR	N/A	HDGC: ↓ IC, CC less extensive than FA	HDGC: No differences	p<0.05 TFCE corrected
Sritharan	2010	12	17	Not included	18	HD: No differences	N/A	N/A	N/A	N/A	N/A	N/A	N/A	p<0.005 Bonferroni corrected
Dominguez	2013	18	28	29	29	PreHD: No differences HD: ↑ in BG	PreHD: No differences HD: ↑ in BG	N/A	N/A	N/A	N/A	N/A	N/A	p<0.05 corrected for multiple comparisons
Hobbs	2015	15	40	Not included	61	HD: No differences	HD: ↑ in BG	HD: ↑ in BG	HD: ↑ in BG	N/A	N/A	N/A	N/A	p<0.05 uncorrected
Gregory	2015	15	36	Not included	48	HD: ↓ in CC, CR, IC	N/A	HD: ↑in CC and IC	HD: ↑in CC	N/A	N/A	N/A	N/A	p<0.05 FDR corrected
Poudel	2015	18	27	28	25	N/A	N/A	N/A	N/A	PreHD: No differences HD: ↓ FA in CC and cingulum	N/A	PreHD: No differences HD: No differences	PreHD: No differences HD: ↑ CC, IC, striatal projections of WM	p<0.05 TFCE corrected
Dominguez	2016	30	36	40	36	PreHD: No differences	PreHD: No differences HD: No	N/A	N/A	N/A	N/A	N/A	N/A	p<0.05 Bonferroni corrected

						HD: ↑ in BG	differences							
Harrington	2016	24	37	67	Not included	PreHD: No differences	PreHD: ↑ in SFOF	PreHD: No differences	PreHD: No differences	N/A	N/A	N/A	N/A	p<0.05 FDR corrected
Sweidan	2020	7	11	Not included	13	HD: ↓ in CC	HD: No differences	N/A	N/A	HD: No differences	HD: No differences	N/A	N/A	p<0.05 TFCE corrected
Pflanz	2020	12	19	Not included	19	PreHD: No differences	PreHD: ↑ in BG	N/A	N/A	PreHD: No differences		N/A	N/A	ROI: p<0.05 Bonferroni corrected Whole brain: p<0.05 TFCE corrected

AD: Axial Diffusivity, AF: Arciform Fibers, ATR: Anterior Thalamic Radiations, CC: Corpus Callosum, CP: Cerebral Peduncles, CR: Corona Radiata, CST: CorticoSpinal Tract, DTI: Diffusion Tensor Imaging, FA: Fractional Anisotropy, IC: Internal Capsule, EC: External Capsule, HD: Huntington’s disease, HDGC, Huntington’s disease gene-carriers, IFOF: Inferior Fronto Occipital Fasciculus, ILF: Inferior Longitudinal Fasciculus, MD: Mean Diffusivity, preHD: Premanifest Huntington’s disease, PTR: Posterior Thalamic Radiations, RD: Radial Diffusivity, SS: Sagittal Strattum, SLF: Superior Longitudinal Fasciculus, SFOF: Superior Fronto Occipital Fasciculus, UNC: uncinate fasciculi
